# Supplementary material for: Conditional control of fluorescent protein degradation by an auxin-dependent nanobody
Source: Nat Commun. 2018 Aug 17;9:3297. doi: 10.1038/s41467-018-05855-5 (PMC6098157; doi:10.1038/s41467-018-05855-5)
Supplement: Supplementary file 1 — Supplementary Information [file 41467_2018_5855_MOESM1_ESM.pdf]

Conditional control of fluorescent protein degradation by an auxin-dependent nanobody

Daniel et al.,

Supplementary Information

Supplementary Figures

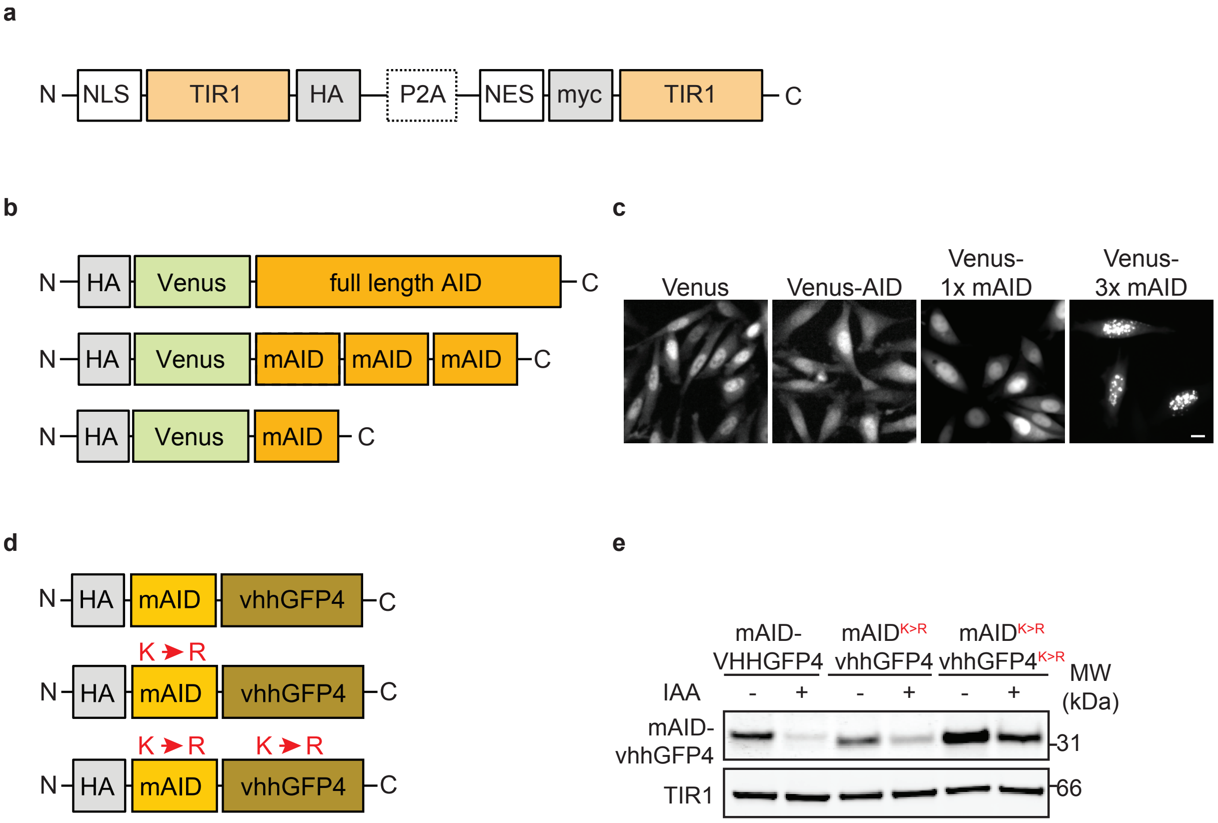

Supplementary Figure 1 | Development of a lysine-less mAID-nanobody to target GFP and GFP-like proteins through the auxin system. (**a**) Schematic representation of the TIR1 construct. (**b**) Schematic representation of full length AID, triple mAID and single mAID reporters and (**c**) their localization in living HeLa cells. Scale bar 10 µm. (**d**) Schematic representation of wildtype and lysine-less mAID-nanobody constructs. (**e**) Western blot analysis of HeLa cells expressing mAID-nanobody constructs illustrated in (d) with or without treatment with 0.5 mM IAA for 180 minutes. TIR1 serves as a loading control.

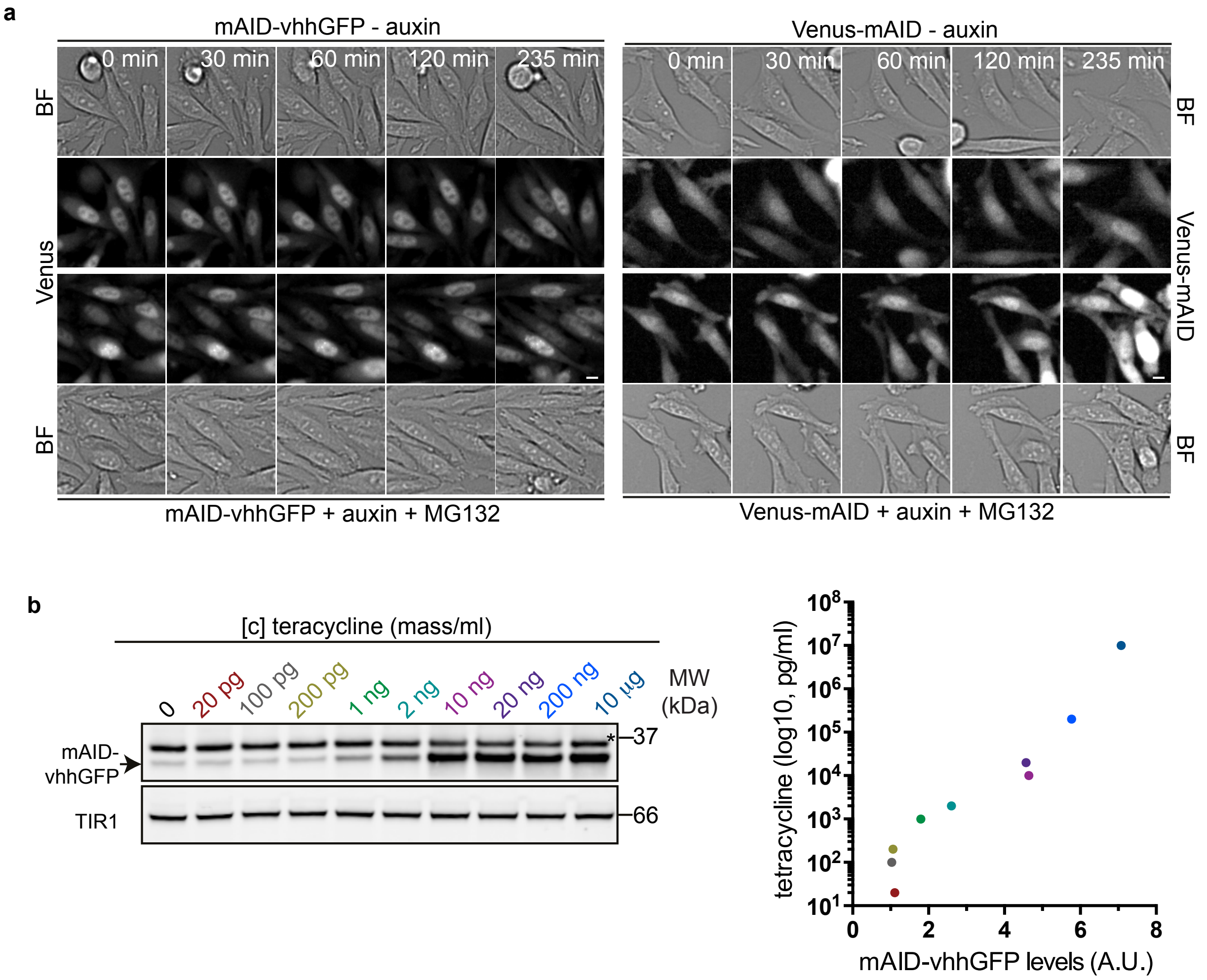

Supplementary Figure 2 | Degradation of Venus by mAID-nanobody and AID systems and titration of mAID-nanobody expression. (**a**) Time-lapse images of Venus targeted by the mAID-nanobody and of Venus-AID without auxin and in the presence of 0.5 mM auxin together with 10 µM of the proteasome inhibitor MG132. Note, the associated images with auxin addition are presented in Fig. 2. (**b**) Quantitative Western blot analysis by near infra-red imaging and quantification of mAID-nanobody expression in response to tetracycline titration for 24 hours in relation to the data shown in Fig. 2d. Arrow indicates the mAID-nanobody, asterisk Venus, which both bear the HA epitopes (see Methods).

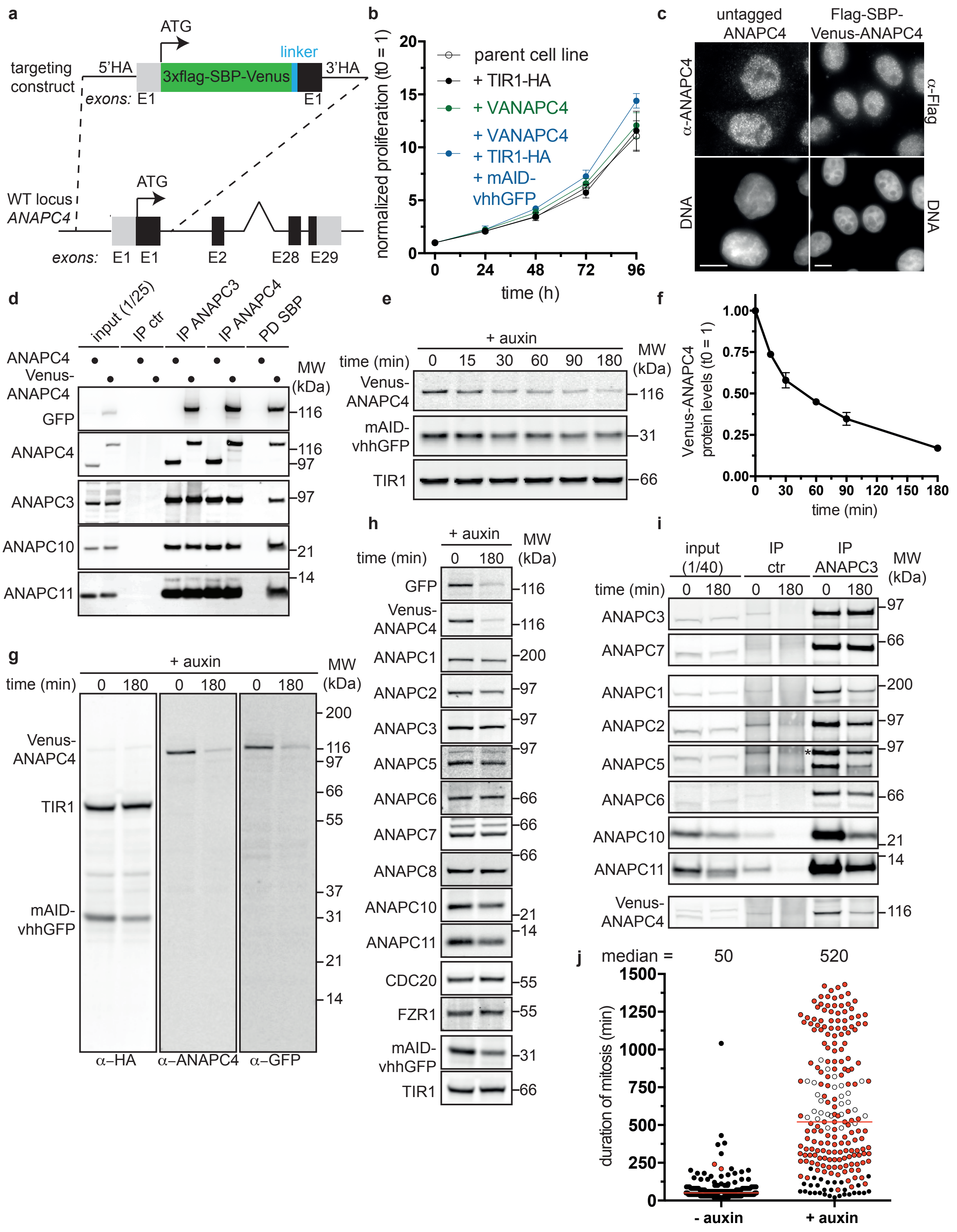

Supplementary Figure 3 | Targeting of the endogenous *ANAPC4* locus and the effect of ANAPC4 depletion by mAID-nanobodies on the APC/C and the duration of mitosis. (**a**) Targeting scheme to insert 3xFlag-SBP-Venus into *ANAPC4* loci by CRISPR/Cas9-assisted homologous recombination.

(continued on next page)

(**b**) Proliferation analysis for the indicated time showing the behavior of the parent HeLa FRT/TO cell line compared to the stable cell lines expressing TIR1, 3xFlag-SBP-Venus-ANAPC4 (VANAPC4) and TIR1 + 3xFlag-SBP-Venus-ANAPC4 + mAID-nanobodies. Data represent mean ± s.d. (n≥4, all conditions). (**c**) Immunostaining of endogenous untagged ANAPC4 and endogenously-tagged 3xFlag-SBP-Venus-ANAPC4. Scale bar: 10 µm. (**d**) Western blot analysis of ANAPC3, ANAPC4 and SBP purifications from parent (ANAPC4) and endogenously tagged (Venus-ANAPC4) cells showing the integration of Venus-ANAPC4 into the APC/C. (**e**) Western blot analysis and quantification (**f**) of Venus-ANAPC4 levels in total lysates after the addition of auxin for the indicated time. Data represent the mean and s.e.m., (n=3), determined by quantitative near infra-red Western blot analysis (**g**) Western blot analysis showing the complete membranes detected with anti-ANAPC4 and anti-GFP antibodies after degradation of Venus-ANAPC4 by addition of 0.5 mM IAA for 3 hours. TIR1 serves as the loading control. (**h**) Western blot analysis showing the stability of other APC/C subunits as well as APC/C co-activators CDC20 and FZR1 after depleting Venus-ANAPC4 with mAID-nanobodies for 3 hours. (**i**) Western blot analysis of control (HA) and ANAPC3 immunoprecipitations assaying APC/C integrity after depleting Venus-ANAPC4. Note, only the association with ANAPC7, a direct binder of ANAPC3 is not affected by degrading Venus-ANAPC4 indicating a partial disassembly of the APC/C holoenzyme. **(j)** Duration of mitosis determined from time-lapse experiments directly after the addition of auxin. Black circles represent cell that finished mitosis normally, open circles represent cells that remained arrested in mitosis during 24 hours of imaging , and red circles indicate cells that underwent mitotic catastrophe after a prolonged mitotic arrest. Red lines represent the median of 202 and 221 cells from – and + auxin treatment, respectively. Note, only cells that entered mitosis within the first 16.6 hours of the experiment were considered for analysis.

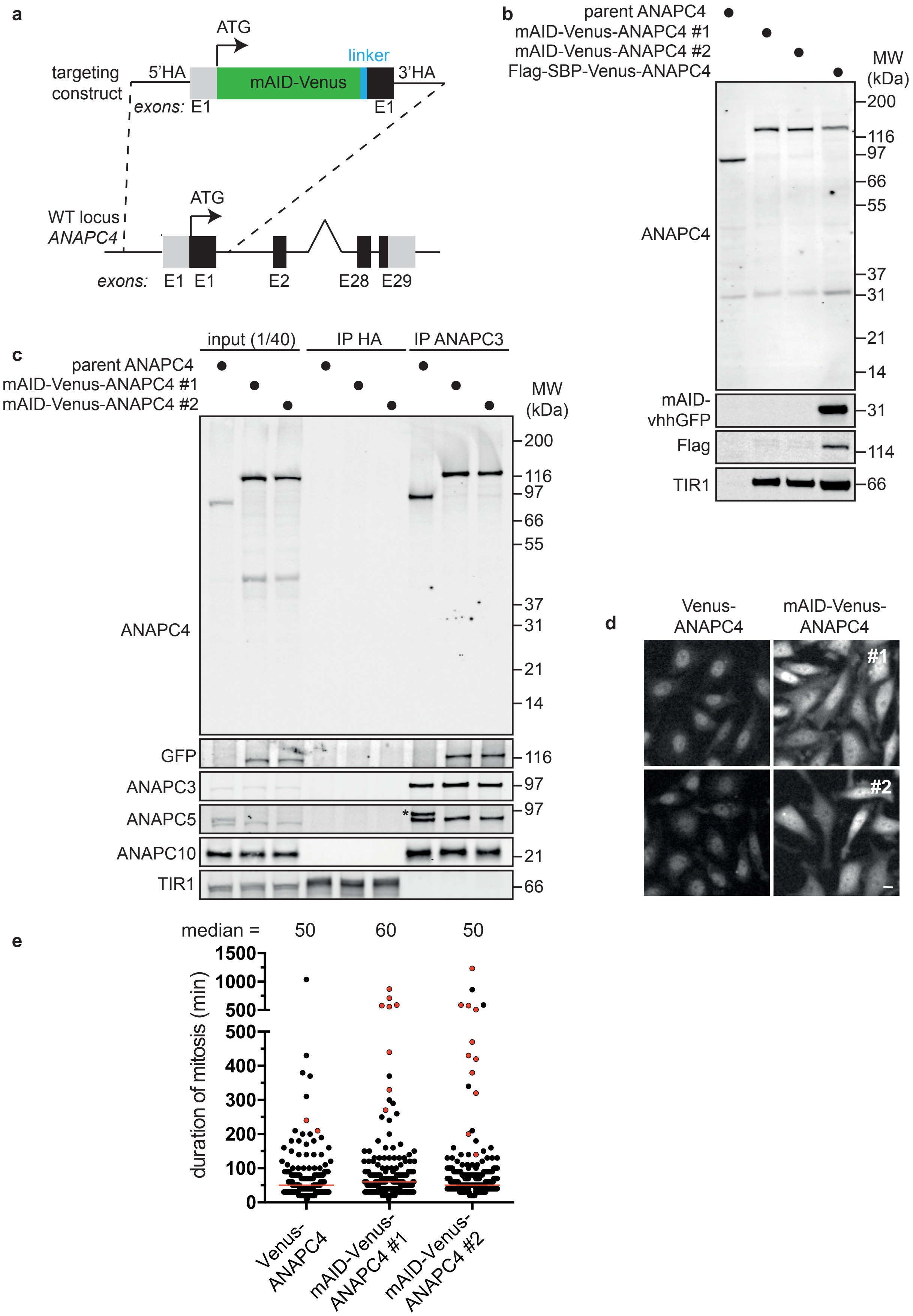

Supplementary Figure 4| Targeting of the endogenous *ANAPC4* locus with AID-Venus.

(Figure legend on next page)

(**a**) Targeting scheme to insert AID-Venus into the *ANAPC4* locus by CRISPR/Cas9-assisted homologous recombination. (**b**) Western blot analysis of total cell lysates comparing the expression and molecular size of parent, Flag-SBP-Venus and AID-Venus-tagged endogenous ANAPC4. (**c**) Western blot analysis of control (HA) and ANAPC3 immunoprecipitation showing AID-Venus-ANAPC4 incorporation into the APC/C and that the tagged allele represents almost all ANAPC4 assembled into the APC/C (see Methods for discussion of the two additional ANAPC4 alleles present in AID-Venus-ANAPC4 cell lines). (**d**) Localization of AID-Venus-ANAPC4 compared to Flag-SBP-Venus-ANAPC4 (Venus-ANAPC4) in living cells. Note, tagging of ANAPC4 with AID-Venus but not tagging with equally sized Flag-SBP-Venus reduces the nuclear enrichment of ANAPC4 compared to untagged ANAPC4 (see Supplementary Fig. 2b). Scale bar: 10 µm. (**e**) Duration of mitosis determined from time-lapse experiments comparing cell lines expressing endogenous Flag-SBP-Venus-ANAPC4 (Venus-ANAPC4) and AID-Venus-ANAPC4. Black circles represent cells that finished mitosis normally and red circles indicate cells that underwent mitotic catastrophe after a prolonged mitotic arrest. Red lines represent the median of 202 cells for Venus-ANAPC4, of 202 cells for AID-mVenus-ANAPC4 clone #1 and of 175 cells for AID-mVenus-ANAPC4 clone #2. Note, the data for Venus-ANAPC4 are reproduced for comparison from the graph shown in Supplementary Figure 3j.

a

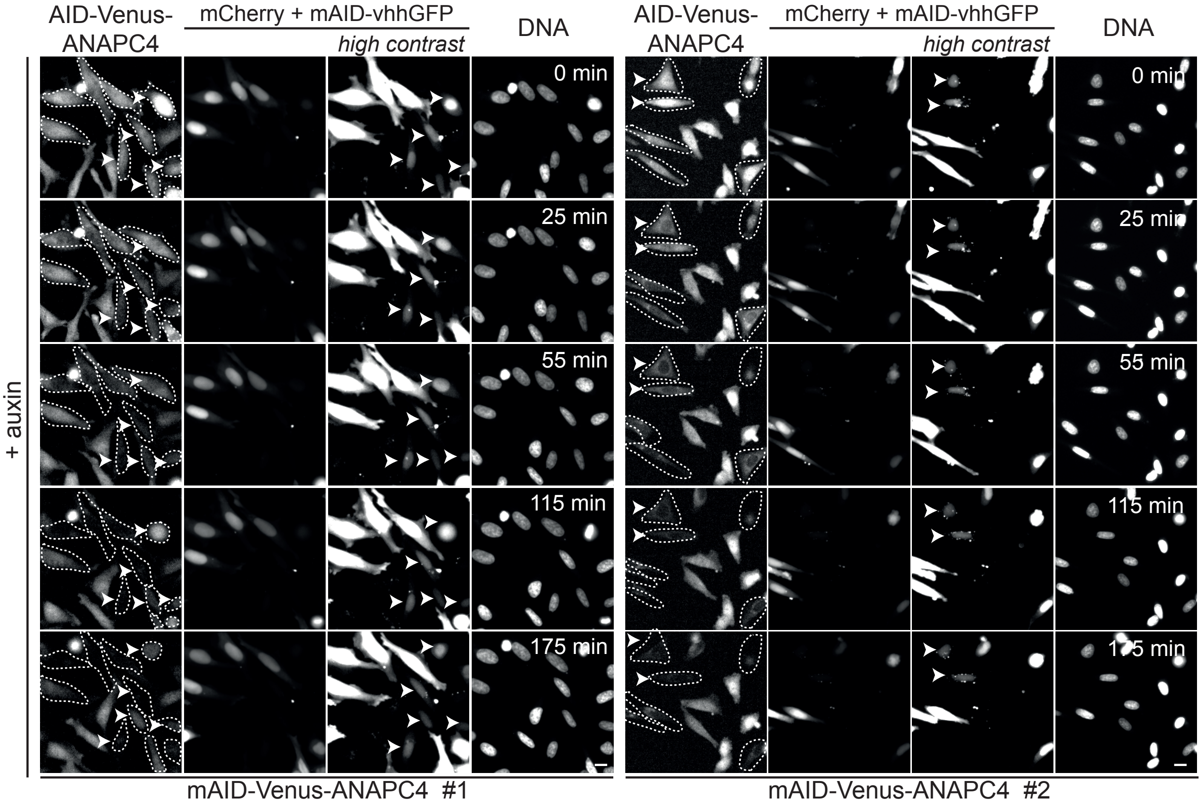

b

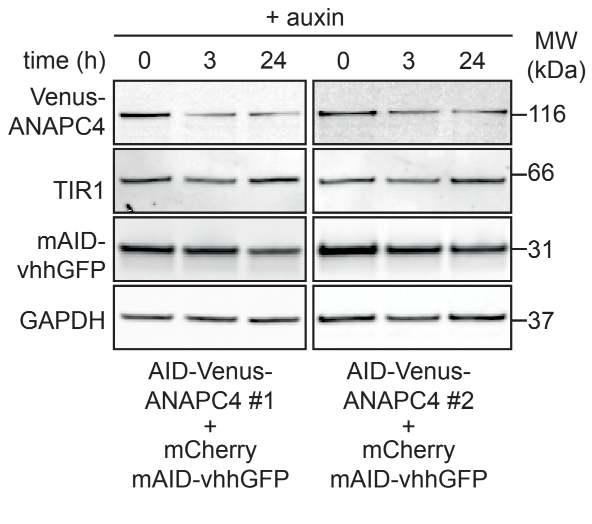

Supplementary Figure 5 | mAID-nanobody expression rescues mAID-Venus-ANAPC4 degradation. (**a**) Time-lapse images showing mAID-Venus-ANACP4 degradation in two clones upon transient transfection with the mAID-nanobody. The mAID-nanobody is expressed from the same mRNA as mCherry, which thereby marks positive cells (dotted lines). Note, degradation occurs in cells with greatly varying levels of mCherry and thus mAID-nanobody expression (see very low mCherry expressing cells marked by arrowhead), Scale 10 µm. (**b**) Western blot analysis of total lysates from cells of two mAID-Venus-ANACP4 clones transiently transfected with mAID-nanobody treated as in (a) with auxin for the indicated time.

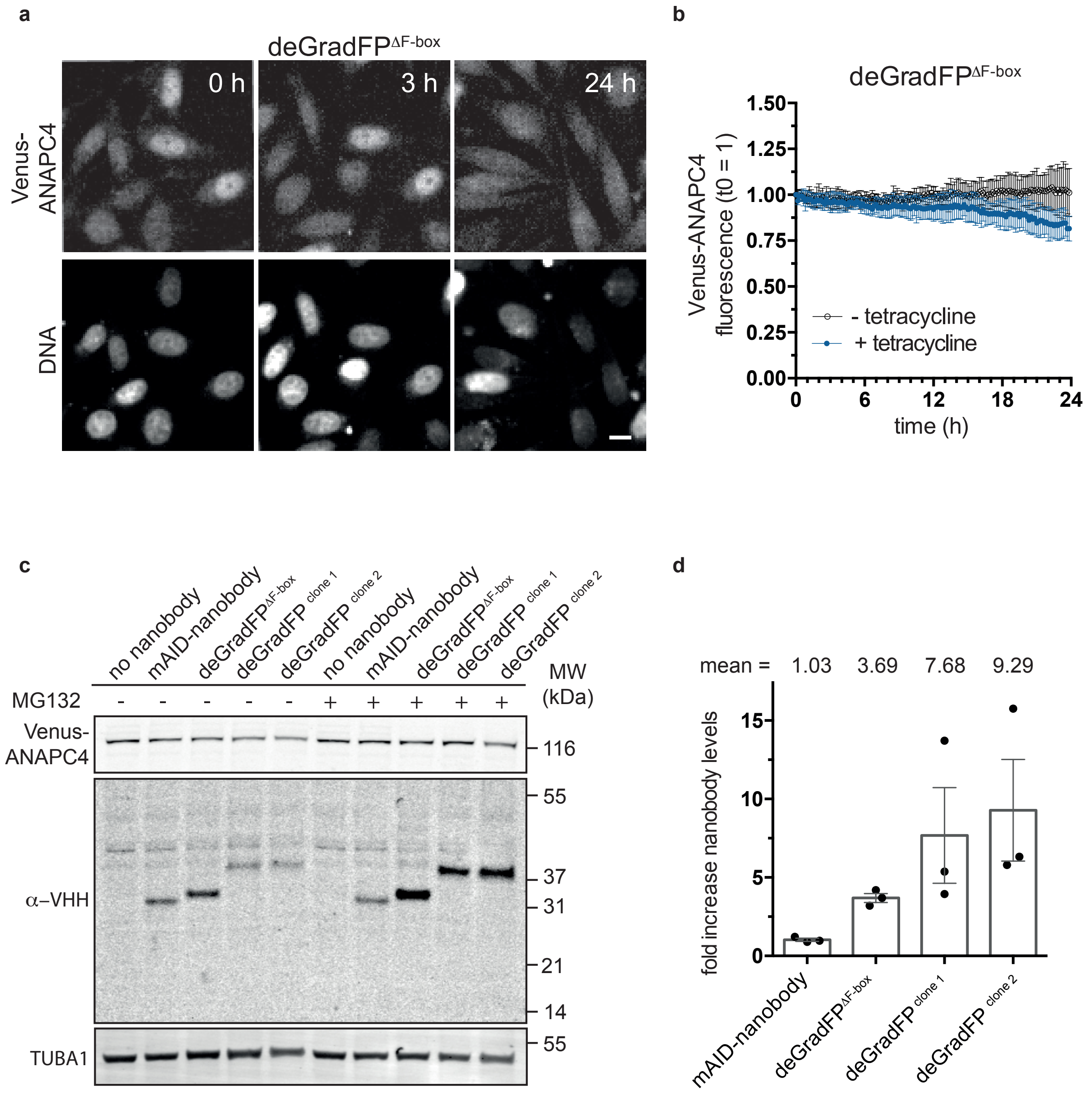

Supplementary Figure 6 | Venus-ANAPC4, deGradFP and deGradFP$^{\Delta Fbox}$ stability. (**a**) Time-lapse images showing Venus fluorescence after induction of deGradFP$^{\Delta Fbox}$ expression with 1 µg/ml tetracycline for the indicated time. The associated images showing degradation of Venus-ANAPC4 by deGradFP are presented in Fig. 5. Scale bar: 10 µm. (**b**) Quantification of time-lapse shown in (a). Data represent mean ± s.d. (n=8, - auxin; n=10, +auxin). (**c**) Quantitative near-infrared Western blot analysis comparing the levels of deGradFP and deGradFP$^{\Delta Fbox}$ expression with and without proteasomal inhibition with 10 µM MG132 for 8 hours. Note, the expression of deGradFP and deGradFP$^{\Delta Fbox}$ was induced 2 hours prior to MG132 addition by 1 µg/ml tetracycline. (**d**) Quantification of the data presented in (c). Data represent mean ± s.e.m. (n=3). Fold increase was calculated by dividing the loading-normalized levels of nanobodies in MG132-treated samples by the loading-normalized levels of samples without MG132.

a

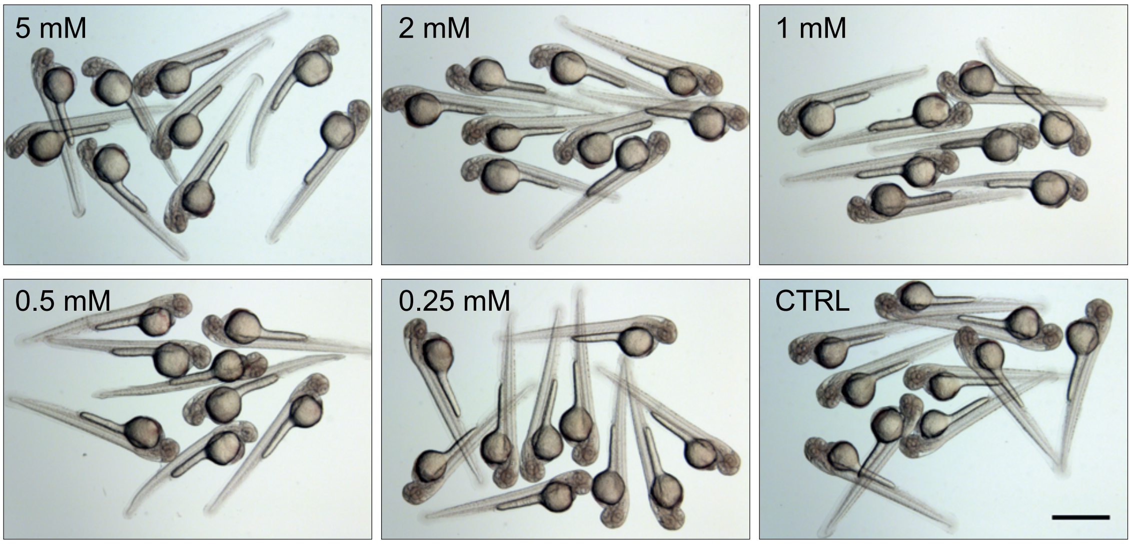

b

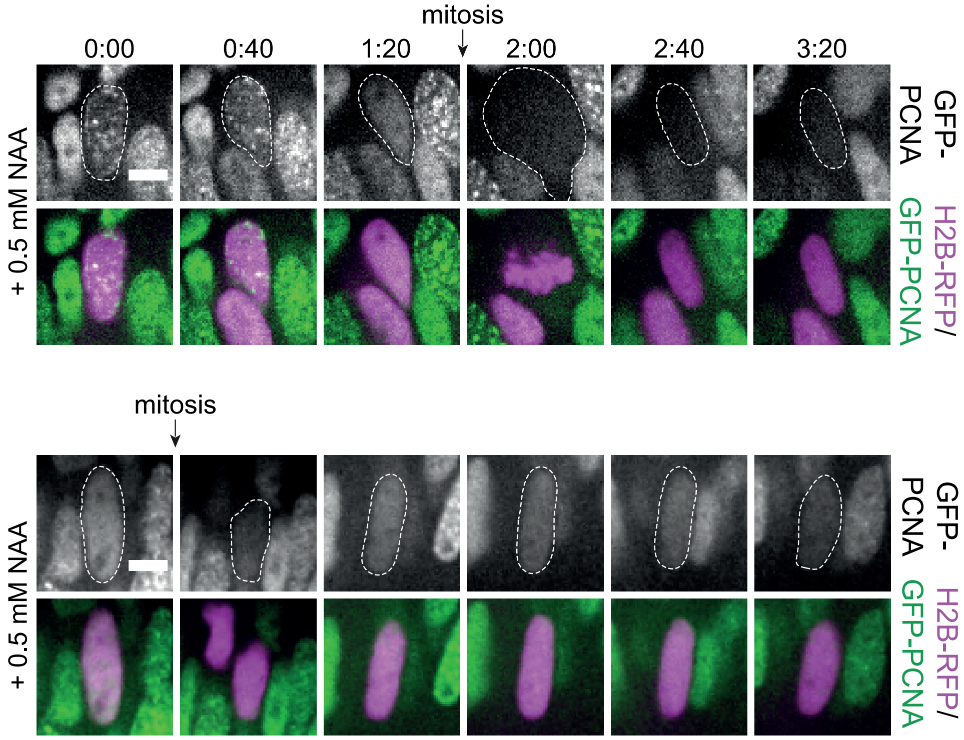

Supplementary Figure 7 | Auxin tolerance and GFP-PCNA degradation in zebrafish. (**a**) Bright field images of zebrafish embryos grown in the presence of the indicated concentration of IAA until 48 hpf. Scale bar: 1 mm. (**b**) Time-lapse imaging of GFP-PCNA degradation in response to 0.5 mM NAA as shown in Fig. 6d. Cells with degradation system are H2B-RFP positive. Scale bar: 5 µm.

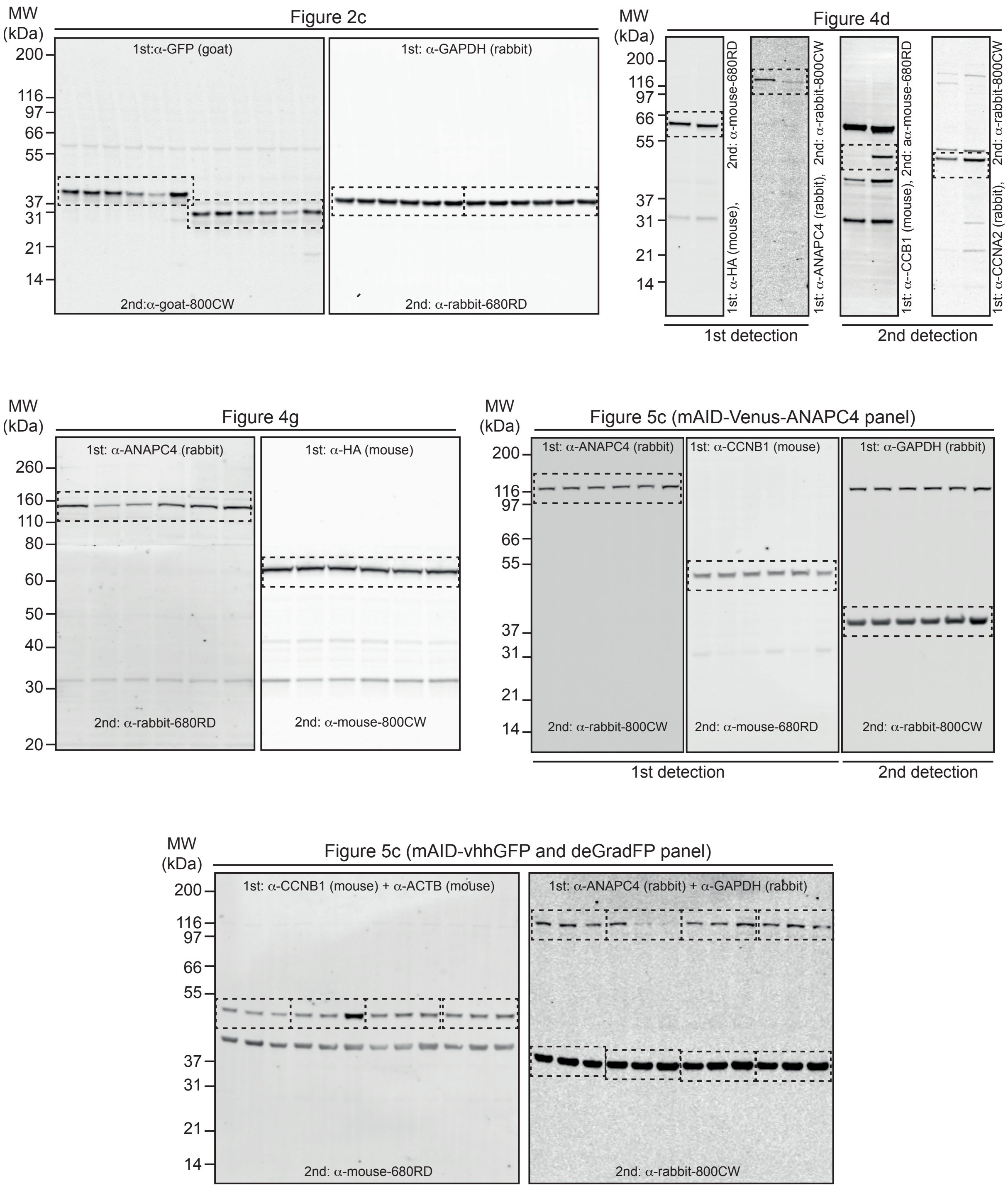

Supplementary Figure 8 | Complete scans of all Western analyses presented in Figures 2, 4 and 5. Cropped regions are indicated by dashed boxes. 680RD and 800CW detections indicate scans of the same blot using secondary antibodies coupled to dyes that are excited by 680 nm or 800 nm light, respectively.
