# Supplementary material for: Conditional control of fluorescent protein degradation by an auxin-dependent nanobody
Source: Nat Commun. 2018 Aug 17;9:3297. doi: 10.1038/s41467-018-05855-5 (PMC6098157; doi:10.1038/s41467-018-05855-5)
Supplement: Supplementary file 3 — Description of Additional Supplementary Files [file 41467_2018_5855_MOESM3_ESM.pdf]

Description of Additional Supplementary Files

File Name: Supplementary Movie 1
Description: Time-lapse imaging of Venus-ANAPC4 cells expressing the mAID-nanobody treated with 0.5 mM auxin at t = 0 to induce degradation. The DNA was stained by SiR-Hoechst. Scale bar: 25 µm.

File Name: Supplementary Movie 2
Description: Comparative degradation of Venus-ANAPC4 and mAID-Venus-ANAPC4 by mAID-nanobody, deGradFP, and the classical auxin systems monitored by time-lapse imaging. Degradation was initiated at t = 0 min, either by the addition of auxin to Venus-ANAPC4 + mAID-vhhGFP and mAID-Venus-ANAPC4 cells or by adding tetracycline to induce the expression of deGradFP.
Scale bar: 50 µM.

File Name: Supplementary Movie 3
Description: mAID-Venus-ANAPC4 is targeted for degradation by the mAID-nanobody. Time-lapse imaging of mAID-Venus-ANAPC4 cells. The degradation mAID-Venus-ANAPC4, which cannot be targeted for degradation by the classical auxin system, is rescued by transient transfection of the mAID-nanobody. 0.5 mM auxin was added at t= 0 min. Scale bar: 50 µM.
